# Circ_0000144 acts as a miR-1178-3p decoy to promote cell malignancy and angiogenesis by increasing YWHAH expression in papillary thyroid cancer

**DOI:** 10.1186/s40463-022-00574-w

**Published:** 2022-07-28

**Authors:** Yinli Ma, Dan Yang, Pingan Guo

**Affiliations:** Department of Inspection, The First People’s Hospital of Fuyang District, No.429, Beihuan Road, Fuyang District, Hangzhou, 311400 Zhejiang China

**Keywords:** PTC, Circ_0,000,144, miR-1178-3p, YWHAH, Angiogenesis

## Abstract

Papillary thyroid cancer (PTC) is the most common subtype of thyroid cancer. Circular RNA hsa_circ_0000144 (circ_0000144) is related to the progression of thyroid cancer. However, the mechanism by which circ_0000144 accelerates PTC progression is still unclear. Circ_0000144 and YWHAH were upregulated in PTC tissues and cells, while miR-1178-3p had an opposite result. Circ_0000144 silencing constrained PTC cell growth in vitro and in vivo and induced apoptosis and repressed migration, invasion, and angiogenesis of PTC cells in vitro. Circ_0000144 acted as a molecular sponge for miR-1178-3p, which targeted YWHAH. MiR-1178-3p inhibitor reversed circ_0000144 silencing-mediated influence on PTC cell malignancy and angiogenesis. Furthermore, YWHAH overexpression overturned miR-1178-3p mimic-mediated influence on malignant behaviors and angiogenesis of PTC cells. Notably, circ_0000144 regulated YWHAH expression by adsorbing miR-1178-3p. Circ_0000144 promoted cell malignancy and angiogenesis by regulating the miR-1178-3p/YWHAH axis in PTC, offering a novel mechanism for the malignancy and angiogenesis of PTC cells.

**Graphical Abstract:**

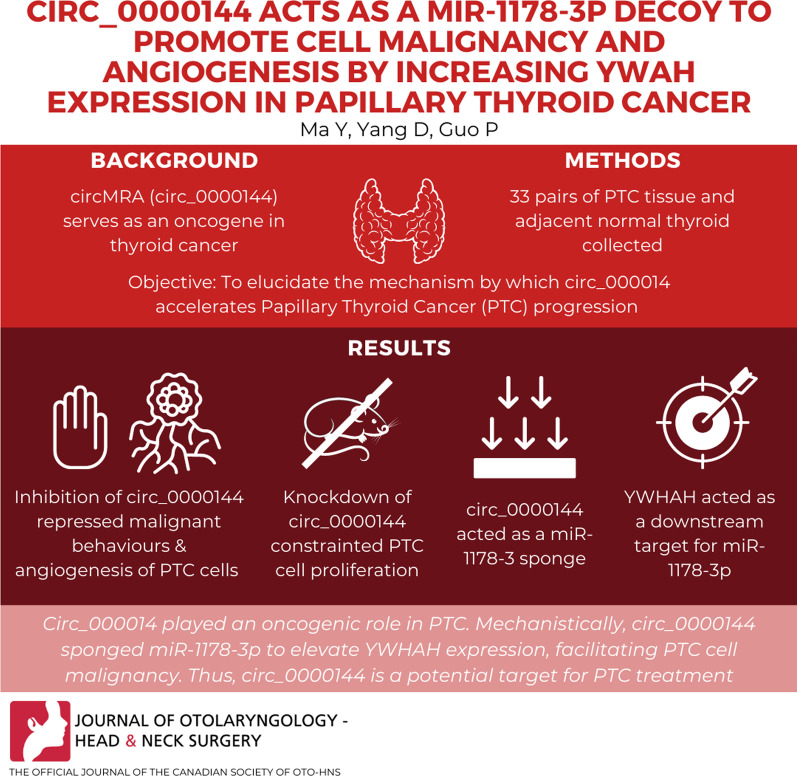

**Supplementary Information:**

The online version contains supplementary material available at 10.1186/s40463-022-00574-w.

## Highlights

1. Inhibition of circ_0000144 repressed malignant behaviors and angiogenesis of PTC cells in vitro.
2. Knockdown of circ_0000144 constrained PTC cell proliferation in vivo.
3. Circ_0000144 acted as a miR-1178-3p sponge.
4. YWHAH acted as a downstream target for miR-1178-3p.

**Supplementary Information:**

The online version contains supplementary material available at 10.1186/s40463-022-00574-w.

## Introduction

Papillary thyroid cancer (PTC), an endocrine malignant tumor, is the most common subtype of thyroid cancer [1, 2]. The growth of PTC is slow, and patients with early PTC have a good prognosis after treatment (thyroidectomy plus radioactive iodine ablation) [3]. However, a small number of PTC patients will progress to more aggressive diseases with recurrence and metastasis [4, 5]. Moreover, the overall 5 years survival rate of PTC is about 97%, but the 5 years survival rate of patients with advanced PTC drops to about 59% [6]. Hence, it is necessary to clarify the mechanism of PTC development to provide directions for the development of new PTC treatment strategies.

Circular RNAs (circRNAs) are mainly derived from the reverse splicing of pre-mRNA exons [7]. Most circRNAs are resistant to RNase R and often show developmental stage or tissue-specific expression [8]. Furthermore, circRNAs disorders are implicated in the occurrence and progression of diseases, such as cardiovascular diseases, diabetes, neurological diseases, and cancer [9]. For instance, circRNA ASH2L facilitated lymphangiogenesis, angiogenesis, and tumorigenesis by upregulating VEGFA in ovarian cancer [10]. CircRNA hsa_circ_0000144 (circ_0000144) is produced by the reverse splicing of the first intron of the SLAMF6 (SLAM Family Member 6) gene. Circ_0000144 has been uncovered to exert an oncogenic role in bladder cancer [11] and gastric cancer [12]. Also, circ_0000144 served as an oncogene in thyroid cancer [13]. Nevertheless, the mechanism by which circ_0000144 accelerates PTC progression has not been entirely interpreted.

A crowd of circRNAs play vital biological functions via serving as microRNA (miR) sponges [14]. MiRs are involved in a lot of developmental and cellular processes in eukaryotic organisms [15]. Recent reports have unmasked that miR-1178-3p exerts a carcinogenic function in nasopharyngeal cancer [16], bladder cancer [17], and pancreatic cancer [18]. However, miR-1178-3p plays an anti-tumor function in PTC [19, 20]. MiRs are vital posttranscriptional modulators that negatively regulate gene expression [21]. The product of the tyrosine 3-monooxygenase/tryptophan 5-monooxygenase activation protein eta (YWHAH) gene belongs to the 14-3-3 protein family, which mediates the protein kinase signaling pathway [22, 23]. Also, YWHAH had been revealed as an underlying diagnostic marker for cholangiocarcinoma [24]. Furthermore, YWHAH acts as an oncogene thyroid cancer [25]. However, the relationship between miR-1178-3p and YWHAH in PTC is unclear.

Herein, we unmasked that circ_0000144 elevated YWHAH expression by acting as a miR-1178-3p decoy and repressing the activity of miR-1178-3p, resulting in accelerating PTC cell malignancy and angiogenesis.

## Materials and methods

### Clinical specimens and ethics statement

33 pairs of PTC tissues and adjacent normal thyroid tissues were collected from patients who underwent surgical resection at the The First People’s Hospital of Fuyang District. All recruited PTC patients signed informed consents. All human-related procedures were conducted in accordance with the Declaration of Helsinki, and the utilization of human PTC tissues was approved by the Ethics Committee of the The First People’s Hospital of Fuyang District. The information on PTC patients was provided in Table 1.

**Table 1:** Association of circ_0000144 expression with clinicopathological factors in PTC patients

Clinicopathological features	Number of cases	Circ_0000144 expression		*P* value
		Low	High	
*Age (years)*				
≤ 45	14	7	7	0.881
> 45	19	10	9	
*Gender*				
Male	13	5	8	0.226
Female	20	12	8	
*Tumor size (cm)*				
≤ 1	15	10	5	0.112
> 1	18	7	11	
*TNM stage*				
I + II	17	12	5	0.024*
III	16	5	11	
*Lymph node metastasis*				
Negative	15	11	4	0.022*
Positive	18	6	12	

*P < 0.05

### Cell culture

Normal human primary thyroid follicular epithelial cells Nthy-ori 3-1, human umbilical vein endothelial cells (HUVECs), and PTC cells TPC-1 and IHH-4 were bought from COBIOER (Nanjing, China) and maintained in a humidified atmosphere at 37 °C with 5% CO_2_. Nthy-ori 3-1 and TPC-1 cells were cultured in Roswell Park Memorial Institute (RPMI)-1640 medium (Thermo Fisher, Waltham, MA, USA) supplemented with 10% FBS (fetal bovine serum) (Sigma, St. Louis, MO, USA), while IHH-4 cells were cultured in a mixture (1:1) of RPMI-1640 (Thermo Fisher) and DMEM (Dulbecco’s modified Eagle’s medium) (Thermo Fisher) supplemented with 10% FBS (Sigma).

### Oligonucleotides and plasmids

Small interference (si) RNA targeting circ_0000144 (si-circ_0000144), negative control (NC) for siRNA (si-NC), short hairpin (sh) RNA against circ_0000144 (sh-circ_0000144), NC for shRNA (sh-NC), miR-1178-3p inhibitor (anti-miR-1178-3p), NC for miR inhibitor (anti-miR-NC), miR-1178-3p mimic (miR-1178-3p), and NC for miR mimic (miR-NC) were synthesized by AoKe Biotech (Beijing, China). Transfection of PTC cells was performed with the Lipofectamine 3000 reagent (Thermo Fisher). The pcDNA-YWHAH (YWHAH) plasmid was constructed using the pcDNA vector (vector) (Addgene, Cambridge, MA, USA).

### Quantitative real-time polymerase chain reaction (RT-qPCR)

Total RNA was extracted using the RNeasy Mini Kit (Qiagen, Redwood, CA, USA). Total RNA (1 μg) was reversely transcribed using the Prime-Script RT reagent kit (TaKaRa, Dalian, China) or TaqMan miRNA Reverse Transcription Kit (Applied Biosystems, Foster City, CA, USA). RT-qPCR was performed using the SYBR Prime Script RT-PCR kit (TaKaRa). All primer sequences were synthesized by AoKe Biotech (Table 2). Glyceraldehyde-3-Phosphate Dehydrogenase (GAPDH) and U6 were used as internal references. Relative expression was figured with the 2^−ΔΔCt^ method. Each experiment was performed in triplicate.

**Table 2:** Primer sequences for RT-qPCR

Genes	Primer sequences (5’-3’)
Circ_0000144	Forward (F): 5’-GAGCAAATTTGGAGCAAAGG-3’
	Reverse (R): 5’-GGGCCTAAGCTAGTCCCTCA-3’
YWHAH	F: 5’-ACGACATGGCCTCCGCTATGAA-3’
	R: 5’-GCTAATGACCCTCCAGGAAGATC-3’
GAPDH	F: 5’-GTCTCCTCTGACTTCAACAGCG-3’
	R: 5’-ACCACCCTGTTGCTGTAGCCAA-3’
miR-1178-3p	F: 5’-GCGCGTTGCTCACTGTTCTT-3’
	R: 5’-AGTGCAGGGTCCGAGGTATT-3’
U6	F: 5’-CTCGCTTCGGCAGCACA-3’
	R: 5’-ACGCTTCACGAATTTGCGTGTC-3’

### Cell counting kit-8 (CCK-8) assay

PTC cells were transfected with specific plasmids and/or oligonucleotides, followed by culturing in 96-well plates at a density of 5 × 10^3^ cells/well for 24, 48, or 72 h. Then, the CCK-8 reagent (10 μL, Dojindo, Kumamoto, Japan) was added and incubated for 1 h. The OD (optical density) value (450 nm) was evaluated using a microplate reader (Bio-TEK, Winooski, VT, USA).

### Cell cycle analysis

The transfected PTC cells (1 × 10^4^) were cultured for 48 h and then detached with 0.025% trypsin (Thermo Fisher). After fixation with 70% ethanol for overnight (4 °C), the cells (1 × 10^6^) were stained with propidium iodide (PI) solution (400 μL), which contained 4 µg/mL PI (Sigma), 0.5 mg/mL RNase A (Thermo Fisher), and 1% Triton X-100 (Sigma). The cellular DNA content was evaluated with a FACS Verse flow cytometer (Becton Dickinson, San Jose, California, USA), followed by analyzing with the FlowJo software (FlowJo v7.6, LLC, Ashland, OR, USA).

### Western blotting

Total protein was extracted using the RIPA buffer containing protease and phosphatase inhibitors (Thermo Fisher). Total protein (30 μg) was isolated with 10% sodium dodecyl sulfate–polyacrylamide gel electrophoresis and then transferred to the polyvinylidene fluoride (PVDF) membrane (Bio-Rad). After sealing with 5% non-fat milk, the membranes were incubated with primary antibodies cyclinD1 (sc-450, 1:200), p21 (sc-53870, 1:200), B-cell lymphoma-2 (Bcl-2) (sc-7382, 1:200), Bcl-2 associated X (Bax) (sc-23959, 1: 200), YWHAH (sc-293464, 1:200), and GAPDH (sc-47724, 1:200). Then, the membranes were incubated with the mouse IgGκ BP-HRP. All primary antibodies were bought from Santa Cruz (Santa Cruz, CA, USA). The blots were developed using the Western Blotting Luminol Reagent (Santa Cruz).

### Cell apoptosis analysis

After culturing for 48 h, the cells were collected, detached, and re-suspended in 1 × binding buffer (1 mL). Subsequently, the cells were stained with the Annexin V-fluorescein isothiocyanate (FITC)/propidium iodide (PI) apoptosis detection kit (Solarbio, Beijing, China) in accordance with the manufacturer’s instructions. The apoptotic rate was determined by the FACS Verse flow cytometer (Becton Dickinson).

### Wound-healing assay

The transfected PTC cells (1 × 10^5^ cells/well) were cultured overnight and then the wounds were made with a sterile pipette tip (200 μL) in the cell monolayer. The wounded gaps were captured with a microscope (Nikon Eclipse E600, Nikon Instruments, Melville, NY, USA) at 0 and 24 h, followed by analyzing with the Image J software (NIH, Bethesda, MD, USA).

### Transwell migration and invasion assays

Transwell chambers with Matrigel (#354,480, Costar, Cambridge, MA, USA) or without Matrigel (#3422, Costar) were used to assess the invasion and migration of transfected PTC cells. The transfected PTC cells (1 × 10^5^) were re-suspended in cell culture medium lacking FBS and then placed on the apical chamber. The cell culture medium encompassing 10% FBS (Sigma) was added to the basolateral chamber. After culturing for 24 h, the migrating and invading cells were fixed with 4% paraformaldehyde (Sigma) and stained with 0.1% crystal violet (Thermo Fisher). The migrating or invading cells in five random fields were counted under the microscope (Nikon Eclipse E600, Nikon Instruments).

### Tube formation assay

HUVECs were placed on 96-well plates, which were pre-cooled, coated with Matrigel (70 μL, Becton Dickinson), and then placed at 37 °C for 30 min. The cells were incubated with the supernatant of PTC cells. The tubule branches were imaged with a microscope (Nikon Eclipse E600, Nikon Instruments), followed by the calculation of the number of tubule branches using the Image J software (NIH).

### Xenograft assay

To obtain TPC-1 cells stable knockdown of circ_0000144, the sequence of sh-circ_0000144 was inserting into the pLKO.1 vector (Thermo Fisher), and sh-NC was utilized as an NC. Then, these plasmids were transfected into HEK293T cells (COBIOER) together with the lentiviral packaging plasmids (Thermo Fisher). Subsequently, TPC-1 cells were infected with lentiviral particles produced by HEK293T cells, followed by selection with puromycin (2 μg/mL) (Sigma).

For the xenograft assay, TPC-1 cells carrying sh-circ_0000144 or sh-NC were subcutaneously injected into the flank of each BALB/c nude mouse (4–6 weeks old, 15–20 g). Tumor volumes were measured once a week. After injection for 4 weeks, the tumor tissues of the euthanized mice were stripped for tumor weight evaluation and subsequent analysis. 10 BALB/c nude mice (Vital River Laboratory, Beijing, China) were divided into 2 groups by random number table. Tumor volume was calculated based on the following equation: Volume = (length × width^2^)/2. The animal experiments were approved by the Animal Ethics Committee of The First People’s Hospital of Fuyang District.

### Immunohistochemistry (IHC) staining

IHC was conducted to detect the proliferative ability of PTC cells in vivo. IHC staining was performed using the Vectastain Universal Elite ABC Kit (Vector Laboratories, Burlingame, CA, USA) based on the manufacturer’s instructions. Paraffin-embedded xenograft tissue Sects. (4 μm thick) were incubated with anti-Ki67 antibody (sc-23900, 1:200, Santa Cruz) at 4 °C for 12 h.

### Dual-luciferase reporter assay

The putative binding sites between circ_0000144 or YWHAH and miR-1178-3p were predicted by the circular RNA interactome or targetscan database. The wild-type (WT) and mutant (MUT) sequences of circ_0000144 and YWHAH 3’ untranslated region (UTR) were synthesized and then inserted into the psiCHECK2 vector (Promega, Madison, WI, USA), respectively. The luciferase activity in PTC cells co-transfected with miR-1178-3p mimic or miR-NC and the luciferase plasmids carrying WT-circ_0000144, MUT-circ_0000144, WT-YWHAH 3’UTR, or MUT-YWHAH 3’UTR was evaluated using a dual-luciferase reporter assay kit (BioVision, Milpitas, CA, USA).

### Statistical analysis

At least three biological repeats were conducted for each experiment, and all data were presented as mean ± standard deviation. GraphPad Prism 7 software (GraphPad, La Jolla, CA, USA) was utilized for statistical analyses. Differences between two or more groups were evaluated using Student’s *t*-test or analysis of variance. All statistical tests were considered significant when *P* < 0.05.

## Results

### Circ_0000144 accelerated PTC cell proliferation and curbed PTC cell apoptosis

**Fig. 1:**
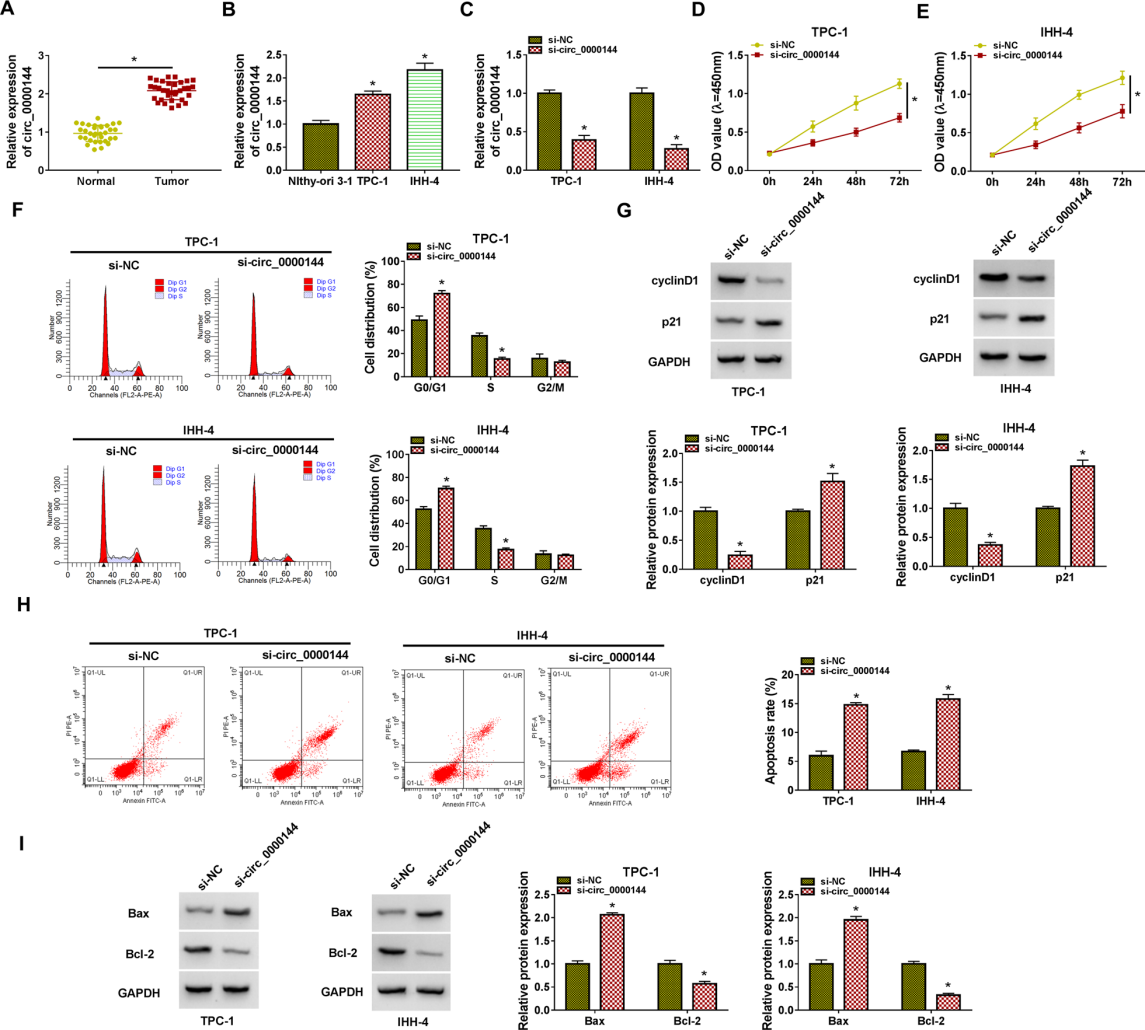
Knockdown of circ_0000144 inhibited cell proliferation and accelerated cell apoptosis in PTC cells. **A** and **B** RT-qPCR revealed the expression of circ_0000144 in PTC tissues and cells. *P < 0.05 vs. adjacent normal thyroid tissues or Nthy-ori 3-1 cells. **C–I** PTC cells were transfected with si-NC or si-circ_0000144. **C** RT-qPCR validated the interference efficiency of si-circ_0000144 in PTC cells. **D**–**F** The proliferation and cell cycle progression of PTC cells were analyzed by CCK-8 and flow cytometry assays. **G** Western blotting detected protein levels of cyclinD1 and p21 in PTC cells. **H** The apoptosis of PTC cells was surveyed by flow cytometry assay. **I** Western blotting analyzed protein levels of Bax and Bcl-2 in PTC cells. *P < 0.05 vs. si-NC

To validate the differential expression of circ_0000144 in PTC, we conducted RT-qPCR analysis. As presented in Fig. 1A, the expression of circ_0000144 in PTC tissues was approximately twice as much as that of adjacent normal thyroid tissues (*P < 0.05). And circ_0000144 expression was associated with TNM stage (*P = 0.024) and lymph node metastasis (*P = 0.022) of PTC patients (Table 1). Also, circ_0000144 was overexpressed in PTC cells (TPC-1 and IHH-4) in contrast to the Nthy-ori 3-1 cells (*P < 0.05) (Fig. 1B). To assess the biological function of circ_0000144 in PTC, we silenced the expression of circ_0000144 in PTC cells. The interference efficiency of si-circ_0000144 in PTC cells was exhibited in Fig. 1C (*P < 0.05). CCK-8 assay exhibited that the transfection of si-circ_0000144 repressed cell proliferation in PTC cells (*P < 0.05) (Fig. 1D and E). Cell cycle analysis exhibited that circ_0000144 inhibition elevated the distribution of PTC cells in the G0/G1 stage and reduced the distribution of PTC cells in the S stage(*P < 0.05) (Fig. 1F). CyclinD1, an important cell cycle regulator, drives cancer cell proliferation [26]. p21 blocks cell cycle progression and exerts a vital role in preventing cell proliferation [27]. As expected, the silence of circ_0000144 reduced the level of cyclinD1 protein and elevated the level of p21 protein in PTC cells (*P < 0.05) (Fig. 1G). Cell apoptosis analysis showed that circ_0000144 knockdown elevated the apoptotic rate of PTC cells (*P < 0.05) (Fig. 1H). Bax is a pro-apoptotic molecule that exerts an inhibitory effect in tumors [28]. Bcl-2, an apoptosis regulator, promotes cell survival by blocking programmed cell death [29]. Also, the level of Bax protein was increased in si-circ_0000144-transfected PTC cells, while the level of Bcl-2 protein had an opposite tendency (*P < 0.05) (Fig. 1I). In sum, these results manifested that circ_0000144 accelerated cell proliferation and repressed cell apoptosis in PTC cells.

### Circ_0000144 facilitated migration, invasion, and induced angiogenesis of PTC cells

**Fig. 2:**
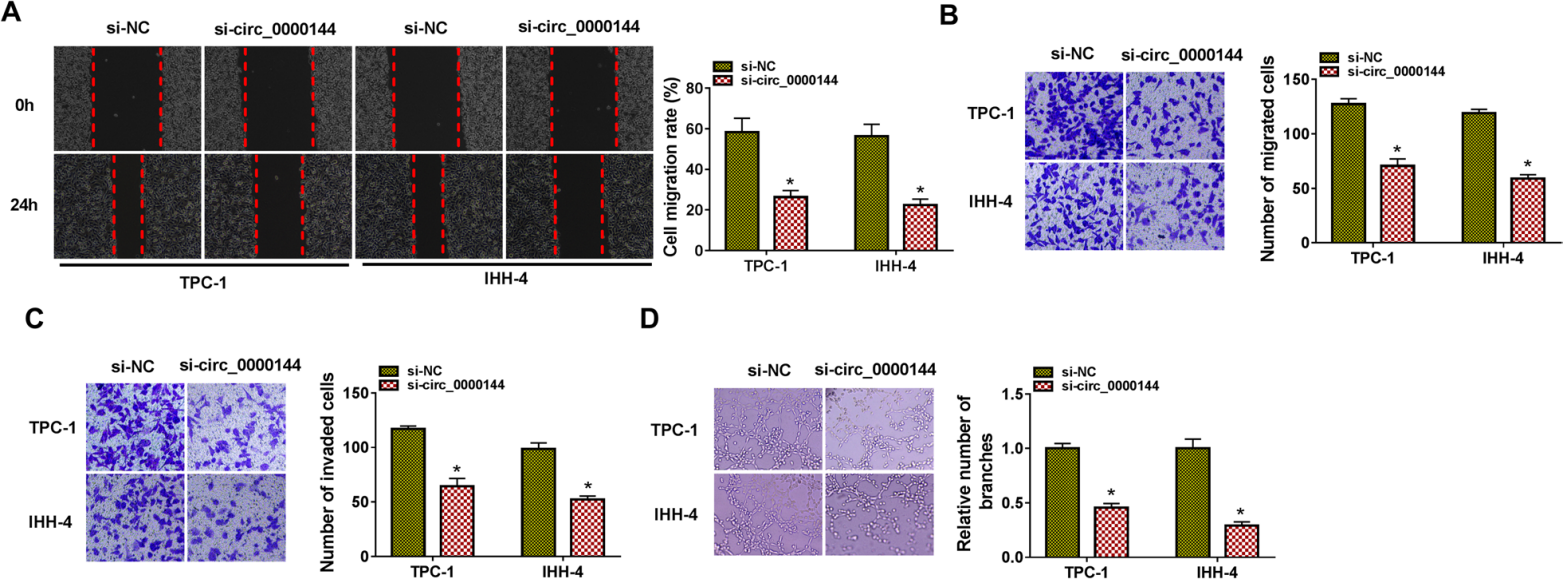
Circ_0000144 knockdown curbed migration, invasion, and angiogenesis of PTC cells. **A**–**D** PTC cells were transfected with si-NC or si-circ_0000144. **A** and **B** Wound-healing and transwell migration assays assessed the migration ability of PTC cells. **C** Transwell invasion assay revealed the invasion of PTC cells. **D** Tube formation assay analyzed the angiogenesis of HUVECs incubated with the supernatant of PTC cells. *P < 0.05 vs. si-NC

Subsequently, we explored the influence of circ_0000144 silencing on migration, invasion, and angiogenesis of PTC cells. Wound-healing and transwell migration assays exhibited that circ_0000144 inhibition reduced the migration ability of PTC cells (*P < 0.05) (Fig. 2A and B). Transwell invasion assay showed that circ_0000144 knockdown repressed the invasion of PTC cells (*P < 0.05) (Fig. 2C). Angiogenesis, the process of vascular remodeling, plays a vital role in tumor growth and metastasis [30]. We then performed the tube formation assay and the results displayed that circ_0000144 silencing repressed tube formation (*P < 0.05) (Fig. 2D). These findings suggested that circ_0000144 silencing repressed migration, invasion, and reduced angiogenesis of PTC cells.

### *Circ_0000144 accelerated xenograft tumor growth *in vivo

**Fig. 3:**
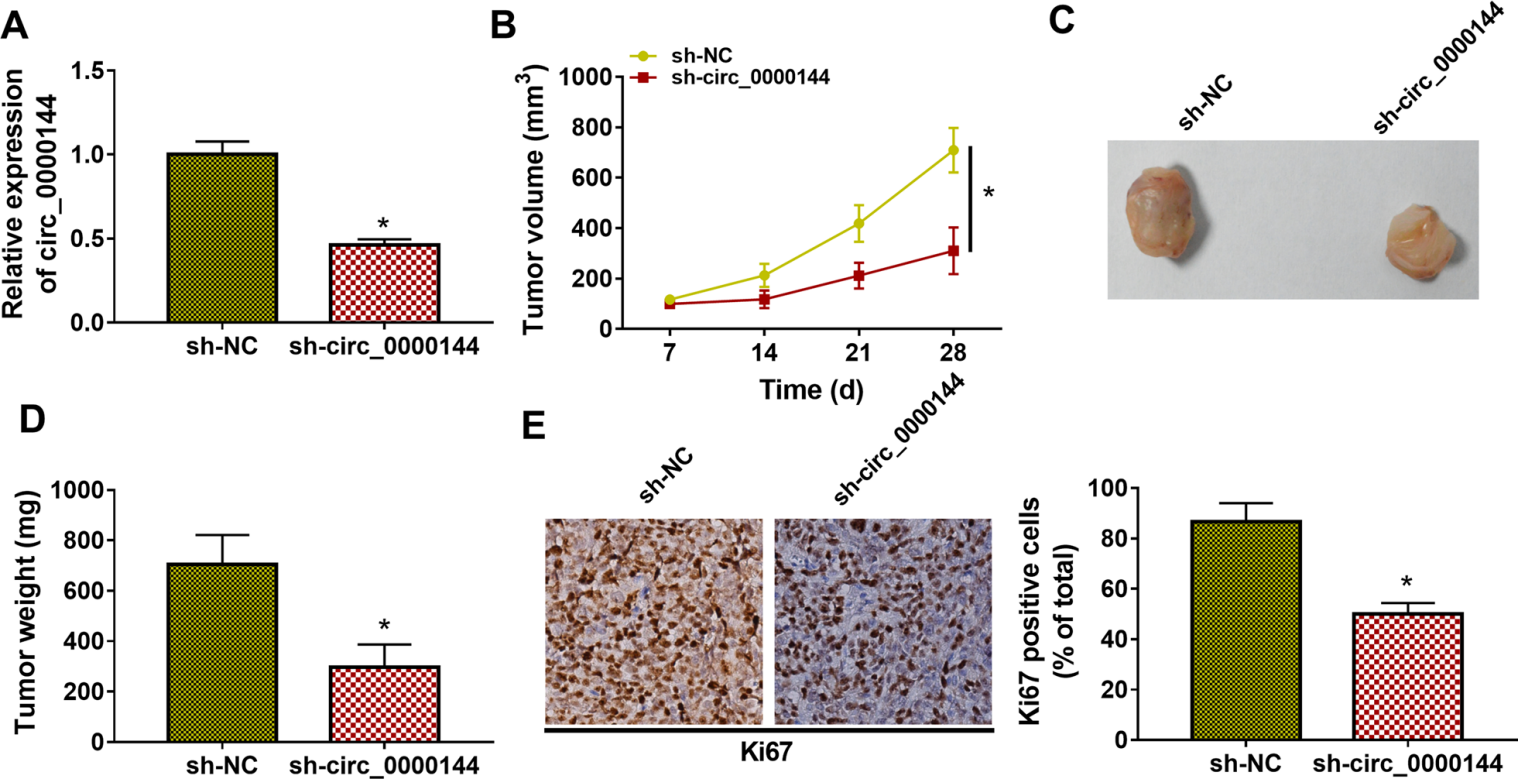
Circ_0000144 promoted PTC cell growth in vivo. **A** RT-qPCR analysis of circ_0000144 expression in xenograft tumors with sh-circ_0000144. *P < 0.05 vs. sh-NC. **B** The volume of xenograft tumors in the sh-circ_0000144 group. *P < 0.05 vs. sh-NC. **C** Representative images of xenograft tumors with sh-circ_0000144 or sh-NC. **D** The weight of xenograft tumors in the sh-circ_0000144 group. *P < 0.05 vs. sh-NC. **E** IHC staining detected the protein level of Ki67 in xenograft tumors with sh-circ_0000144 or sh-NC

The biological function of circ_0000144 in PTC was further validated in vivo through injecting TPC-1 cells stably expressing sh-circ_0000144 or sh-NC into BALB/c nude mice. We observed that the expression of circ_0000144 was observably lower in xenograft tumors with sh-circ_0000144 than those of xenograft tumors with sh-NC (*P < 0.05) (Fig. 3A). Moreover, the volume and weight of xenograft tumors in the sh-circ_0000144 group were apparently lower than those in the sh-NC group (*P < 0.05) (Fig. 3B–D). Ki-67 protein is used as a proliferation marker of tumor cells [31]. IHC staining exhibited that the number of Ki67-positive cells was significantly lower in xenograft tumors with sh-circ_0000144 than that of xenograft tumors with sh-NC (*P < 0.05) (Fig. 3E). These results indicated that circ_0000144 exerted a promoting influence on the proliferation of PTC cells.

### Circ_0000144 acted as a miR-1178-3p sponge

**Fig. 4:**
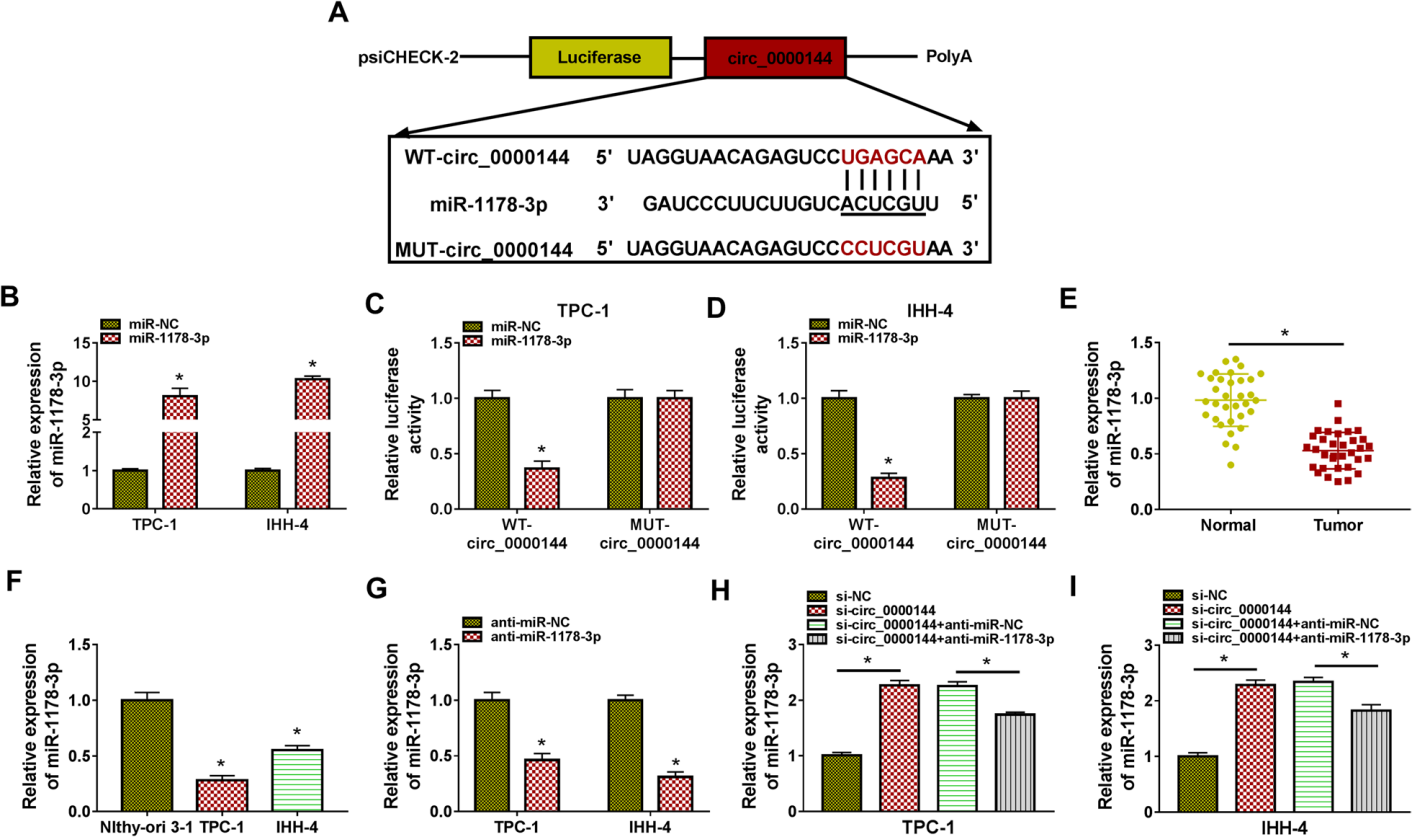
Circ_0000144 was verified as a decoy for miR-1178-3p. **A** A schematic drawing displayed the putative binding sites between circ_0000144 and miR-1178-3p. **B** RT-qPCR analysis of miR-1178-3p expression in PTC cells transfected with miR-1178-3p mimic. *P < 0.05 vs. miR-NC. **C** and **D** Dual-luciferase reporter assay verified the relationship between miR-1178-3p and circ_000014. **E** and **F** RT-qPCR detected the expression of miR-1178-3p in PTC tissues and cells. *P < 0.05 vs. adjacent normal thyroid tissues or Nthy-ori 3-1 cells. **G** The interference efficiency of anti-miR-1178-3p in PTC cells was verified by RT-qPCR. *P < 0.05 vs. anti-miR-NC. **H** and **I** Effect of miR-1178-3p silencing on the expression of miR-1178-3p in si-circ_0000144-transfetced PTC cells was analyzed by RT-qPCR. *P < 0.05 vs. si-NC or si-circ_0000144 + anti-miR-NC

Since circRNA function as a sponge for miRs, we first sought for miRs that might bind to circ_0000144. The circular RNA interactome tool was used to predict miRs that might interact with circ_0000144. Through literature review, 3 miRs (miR-1178-3p, miR-485-3p, and miR-554) associated with PTC and less reported were selected for further analysis. RT-qPCR showed that circ_0000144 knockdown elevated miR-1178-3p and miR-554 expression levels in PTC cells, the effect on the expression of miR-1178-3p (Additional file 1: Fig. S1A and B). So the interaction between miR-1178-3p and circ_0000144 was further explored. As exhibited in Fig. 4A, the sequence of WT-circ_0000144 possessed some bases complementary to miR-1178-3p. To verify the relationship between circ_0000144 and miR-1178-3p, we constructed the luciferase reporter carrying WT-circ_0000144 or MUT-circ_0000144 (Fig. 4A). We overexpressed miR-1178-3p using a mimic and achieved about tenfold overexpression (*P < 0.05) (Fig. 4B). Furthermore, miR-1178-3p mimic decreased the luciferase activity of the WT-circ_0000144 reporter in PTC cells, but there was no observable change in the MUT-circ_0000144 reporter (*P < 0.05) (Fig. 4C and D). RT-qPCR revealed that miR-1178-3p was lowly expressed in PTC tissues and cells (*P < 0.05) (Fig. 4E and F). We also knocked down miR-1178-3p using an inhibitor (*P < 0.05) (Fig. 4G). Additionally, the elevated expression of miR-1178-3p in PTC cells mediated by circ_0000144 inhibition was partly counteracted after miR-1178-3p knockdown (*P < 0.05) (Fig. 4H and I). Collectively, these results suggested that circ_0000144 acted as a decoy for miR-1178-3p in PTC cells.

### Circ_0000144 regulated PTC cell proliferation, apoptosis, migration, invasion, and PTC cell-induced angiogenesis by sponging miR-1178-3p

**Fig. 5:**
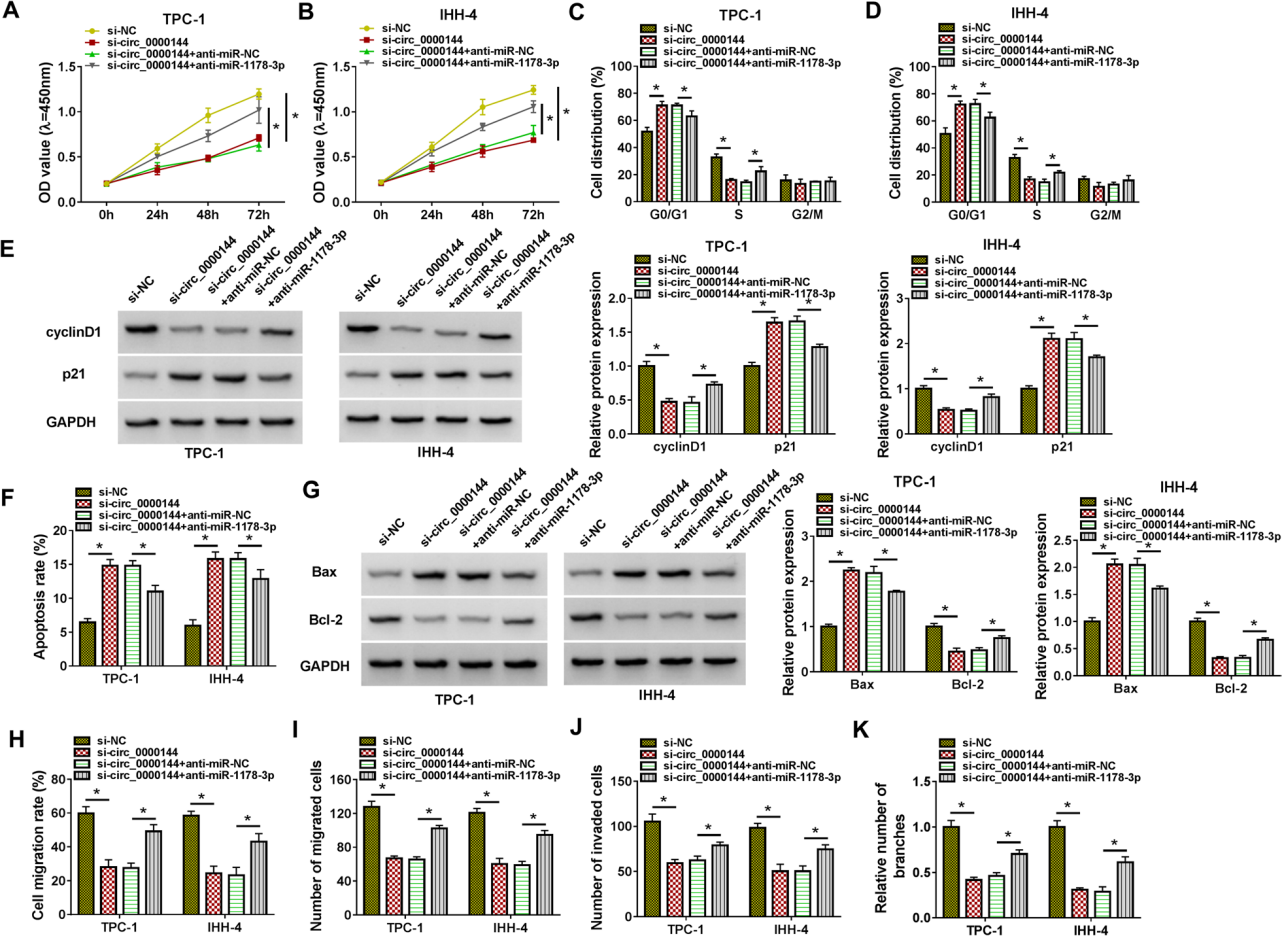
Circ_0000144 sponged miR-1178-3p to regulate PTC cell proliferation, apoptosis, migration, invasion, and angiogenesis. **A**–**K** PTC cells were transfected with si-NC, si-circ_0000144, si-circ_0000144 + anti-miR-NC, or si-circ_0000144 + anti-miR-1178-3p. **A**–**D** CCK-8 and flow cytometry assays were conducted to analyze the proliferation and cell cycle progression of PTC cells. **E** Western blotting analyzed protein levels of cyclinD1 and p21 in PTC cells. **F** Flow cytometry assay detected the apoptotic rate of PTC cells. **G** Western blotting assessed protein levels of Bax and Bcl-2 in PTC cells. **H**–**K** The migration, invasion, and angiogenesis of PTC cells were determined by wound-healing, transwell, and tube formation assays. *P < 0.05 vs. si-NC or si-circ_0000144 + anti-miR-NC

Considering the relationship between circ_0000144 and miR-1178-3p in PTC cells, we further surveyed whether circ_0000144 exerted its function by sponging miR-1178-3p. Rescue experiments exhibited that the introduction of anti-miR-1178-3p partly counteracted the inhibitory impact of circ_0000144 silencing on proliferation and cell cycle progression of PTC cells (*P < 0.05) (Fig. 5A–D). Moreover, miR-1178-3p inhibitor reversed circ_0000144 knockdown-mediated effects on protein levels of cyclinD1 and p21 in PTC cells (*P < 0.05) (Fig. 5E). Furthermore, miR-1178-3p silencing partially overturned the promoting influence of circ_0000144 knockdown on the apoptosis of PTC cells (*P < 0.05) (Fig. 5F). As expected, the upregulation of Bax and the downregulation of Bcl-2 in si-circ_0000144-transfected PTC cells were restored after anti-miR-1178-3p introduction (*P < 0.05) (Fig. 5G). Additionally, the repressive influence of circ_0000144 knockdown on migration, invasion, and angiogenesis was reversed by miR-1178-3p silencing (*P < 0.05) (Fig. 5H–K). Together, these results suggested that circ_0000144 constrained apoptosis and facilitated proliferation, migration, invasion, and angiogenesis of PTC cells by adsorbing miR-1178-3p.

### YWHAH acted as a target for miR-1178-3p

**Fig. 6:**
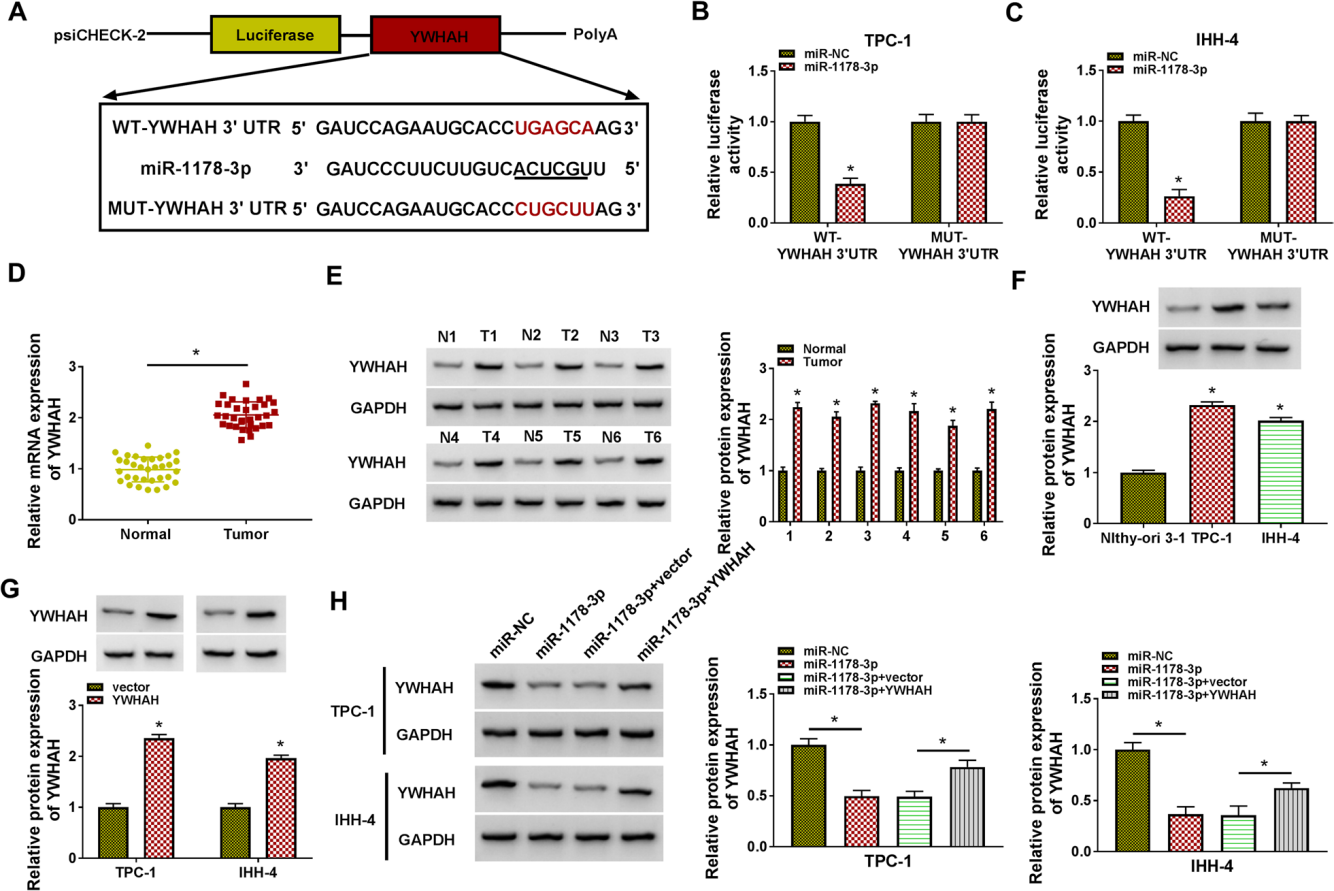
MiR-1178-3p directly targeted YWHAH in PTC cells. **A** A schematic presented the putative binding sites between YWHAH 3’UTR and miR-1178-3p. **B** and **C** The putative binding sites between YWHAH 3’UTR and miR-1178-3p was validated by dual-luciferase reporter assay. *P < 0.05 vs. miR-NC. **D** RT-qPCR analysis of the level of YWHAH mRNA in PTC tissues. *P < 0.05 vs. adjacent normal thyroid tissues. **E** and **F** Western blotting analysis of the protein level of YWHAH in PTC tissues (n = 6) and cells. *P < 0.05 vs. adjacent normal thyroid tissues or Nthy-ori 3-1 cells. **G** Western blotting verified the overexpression efficiency of YWHAH in PTC cells. *P < 0.05 vs. vector. **H** Western blotting analyzed the impact of YWHAH overexpression on the protein level of YWHAH in miR-1178-3p-elelvated PTC cells. *P < 0.05 vs. miR-NC or miR-1178-3p + vector

The targetscan tool was used to predict downstream targets of miR-1178-3p. Five genes (DUSP4, YWHAH, IL17RD, PDPK1, and USP22) associated with PTC and less reported were selected for further analysis after reviewing the relevant literature. We observed that miR-1178-3p overexpression markedly reduced YWHAH and PDPK1 mRNA levels in both TPC-1 and IHH-4 cell lines, and the trend of YWHAH alteration was larger. So, we established the luciferase reporter carrying WT-YWHAH 3’UTR or MUT-YWHAH 3’UTR to further verify their relationship (Fig. 6A). Also, the luciferase activity of the WT-YWHAH 3’UTR reporter was repressed in PTC cells in the existence of miR-1178-3p mimic, whereas the luciferase activity of the MUT-YWHAH 3’UTR reporter did not change (*P < 0.05) (Fig. 6B and C). We also observed that the levels of YWHAH mRNA and protein were upregulated in PTC tissues (*P < 0.05) (Fig. 6D and E). Congruously, the level of YWHAH protein was highly expressed in PTC cells (*P < 0.05) (Fig. 6F). The overexpression efficiency of YWHAH in PTC cells was shown in Fig. 6G (*P < 0.05). In addition, miR-1178-3p mimic suppressed the level of YWHAH protein in PTC cells, whereas this repression was overturned after YWHAH overexpression (*P < 0.05) (Fig. 6H). Collectively, these findings suggested that miR-1178-3p directly targeted YWHAH in PTC cells.

### MiR-1178-3p targeted YWHAH to regulate PTC cell proliferation, apoptosis, migration, invasion, and PTC cell-induced angiogenesis

**Fig. 7:**
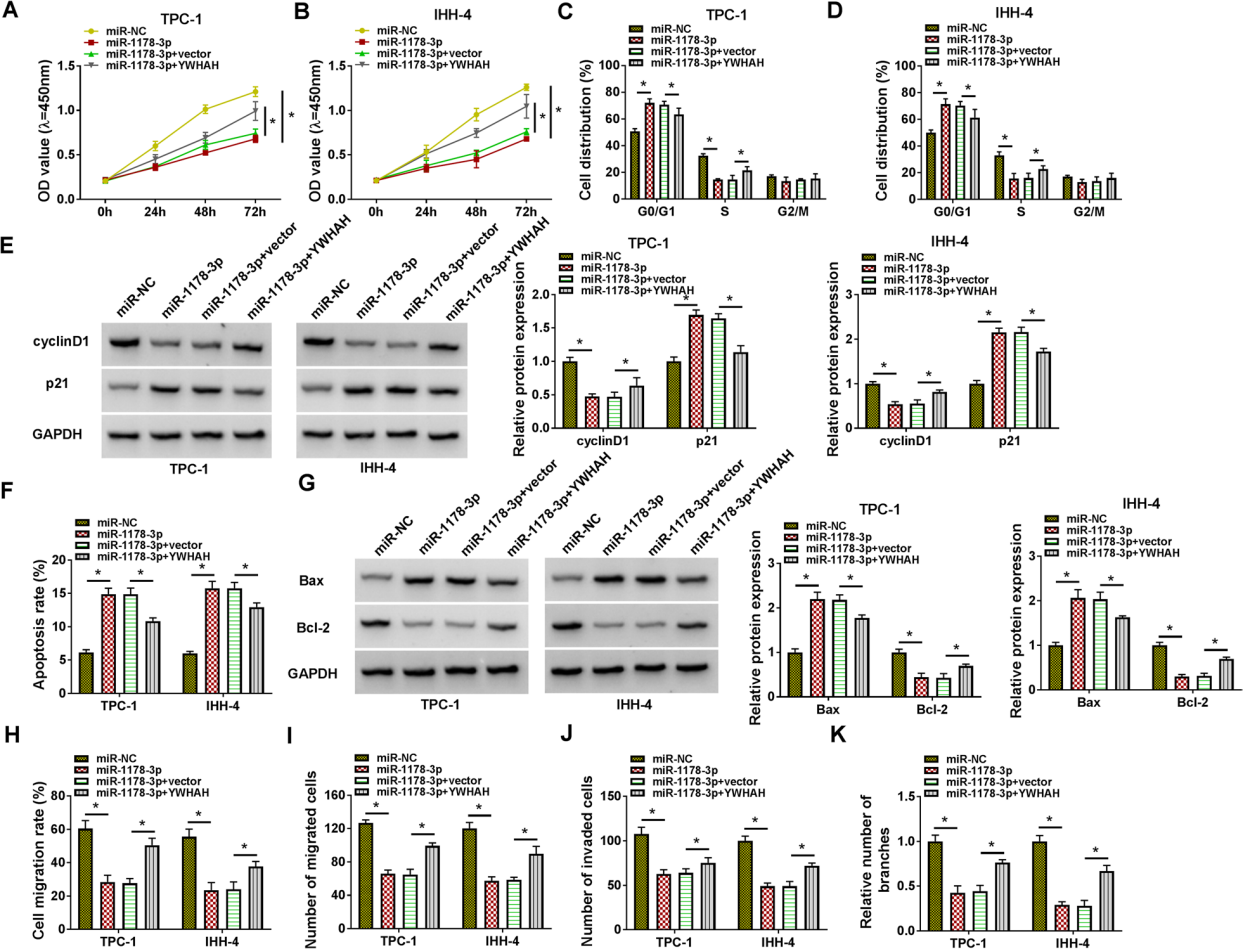
MiR-1178-3p targeted YWHAH to regulate proliferation, apoptosis, migration, invasion, and angiogenesis of PTC cells. **A**–**K** PTC cells were transfected with miR-NC, miR-1178-3p, miR-1178-3p + vector, or miR-1178-3p + YWHAH. **A**–**D** Analysis of proliferation and cell cycle progression of PTC cells by CCK-8 and flow cytometry assays. **E** Assessment of protein levels of cyclinD1 and p21 in PTC cells by western blotting. **F** Detection of the apoptosis of PTC cells by flow cytometry assay. **G** Measurement of protein levels of Bax and Bcl-2 in PTC cells by western blotting. **H**–**K** Analysis of migration, invasion, and angiogenesis of PTC cells by wound-healing, transwell, and tube formation assays. *P < 0.05 vs. miR-NC or miR-1178-3p + vector

To investigate whether miR-1178-3p exerted a repressive role in PTC by targeting YWHAH, we carried out rescue experiments. The results presented that miR-1178-3p mimic constrained the proliferation and cell cycle progression of PTC cells, whereas this inhibition was overturned by the forcing expression of YWHAH (*P < 0.05) (Fig. 7A–D). Consistently, forced YWHAH expression restored the downregulation of cyclinD1 and the upregulation of p21 in PTC cells caused by YWHAH overexpression (*P < 0.05) (Fig. 7E). Also, YWHAH upregulation reversed the elevation of apoptotic rate of PTC cells caused by miR-1178-3p overexpression (*P < 0.05) (Fig. 7F). Moreover, miR-1178-3p mimic reduced the level of Bcl-2 protein and elevated the level of Bax protein in PTC cells, but these trends were restored after YWHAH overexpression (*P < 0.05) (Fig. 7G). In addition, the suppressive effect of miR-1178-3p mimic on migration, invasion, and angiogenesis was offset by YWHAH elevation (*P < 0.05) (Fig. 7H–K). In sum, these results manifested that miR-1178-3p regulated proliferation, apoptosis, migration, invasion, and angiogenesis of PTC cells by targeting YWHAH.

### Circ_0000144 sponged miR-1178-3p to regulate YWHAH expression

**Fig. 8:**
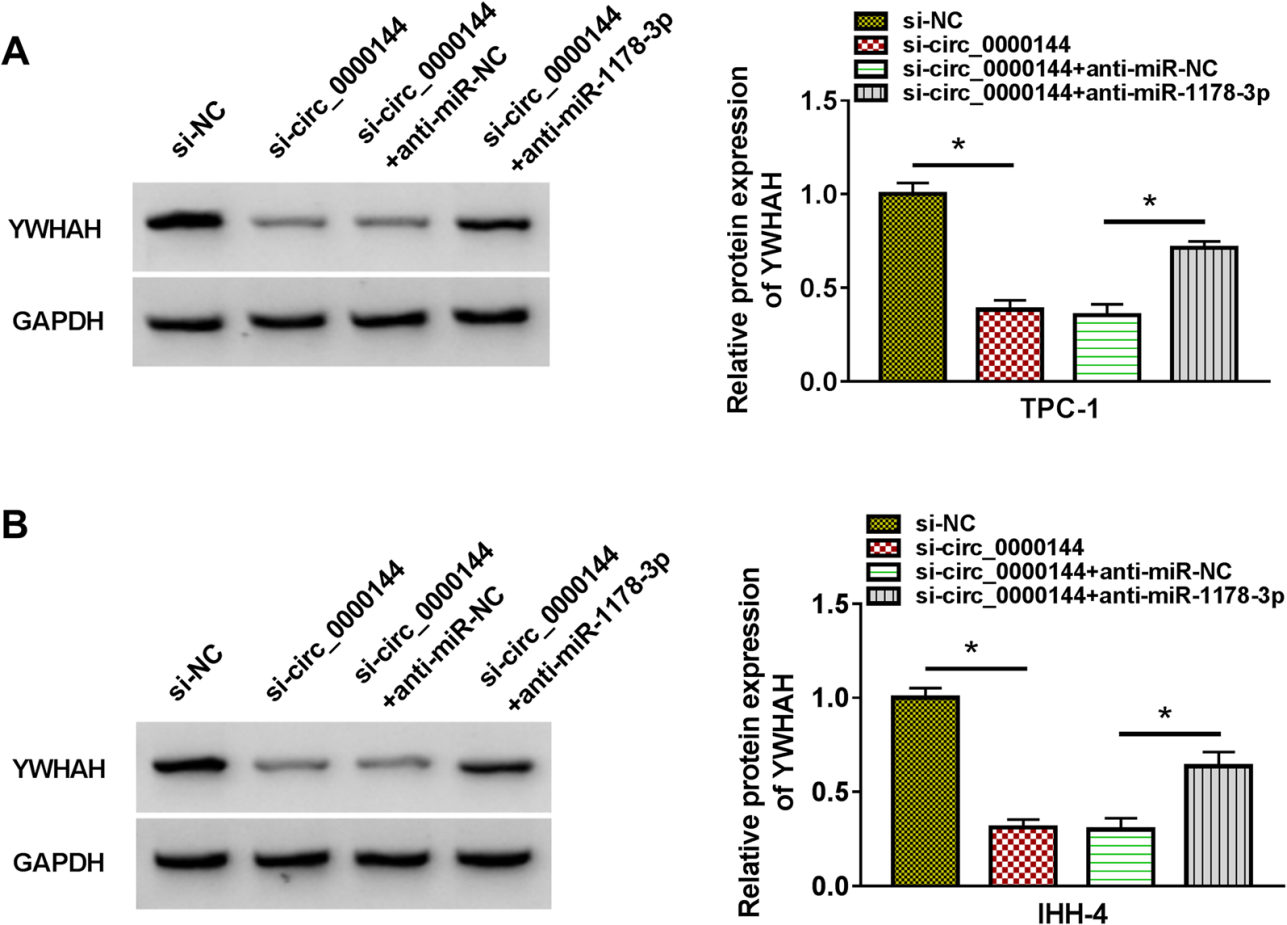
Circ_0000144 adsorbed miR-1178-3p to regulate YWHAH expression. **A** and **B** Western blotting assessed the protein level of YWHAH in PTC cells transfected with si-NC, si-circ_0000144, si-circ_0000144 + anti-miR-NC, or si-circ_0000144 + anti-miR-1178-3p. *P < 0.05 vs. si-NC or si-circ_0000144 + anti-miR-NC

Given that YWHAH shared miR response element with circ_0000144, we further explored whether circ_0000144 played its oncogenic influence by regulating YWHAH expression via adsorbing miR-1178-3p. The results exhibited that circ_0000144 knockdown reduced the level of YWHAH protein in PTC cells, while this decrease was restored after miR-1178-3p inhibition (*P < 0.05) (Fig. 8A and B). These results manifested that circ_0000144 functioned as a miR-1178-3p decoy and regulated YWHAH expression by sponging miR-1178-3p in PTC cells.

## Discussion

The differential expression of circRNAs is observably related to the TNM staging, distant metastasis, and other clinical characteristics of many cancers [32]. Moreover, circRNAs have become a research hotspot to clarify the mechanism of cancer advancement [33]. In the current report, we verified that circ_0000144 adsorbed miR-1178-3p to increase YWHAH expression, resulting in promoting PTC progression.

Herein, circ_0000144 silencing decreased the protein levels of cyclinD1 and Bcl-2 and elevated the protein levels of p21 and Bax, manifesting that circ_0000144 silencing repressed PTC cell proliferation and promoted PTC cell apoptosis in vitro. Also, circ_0000144 silencing repressed PTC migration, invasion, and PTC cell-induced angiogenesis in vitro and curbed PTC cell proliferation in vivo. Circ_0000144 had been uncovered to accelerate malignant behaviors of thyroid cancer via elevating AKT3 expression by adsorbing miR-217 [13]. The similarity was that our results were consistent with the report of Fan et al. [13] regarding the oncogenic role of circ_0000144 in thyroid cancer, but we focused on PTC and performed studies mainly with PTC samples and cell lines. In addition, Fan et al. [13] only explored the function of circ_0000144 in vitro, but we further explored the function of circ_0000144 through an animal xenograft model. Thus, we inferred that circ_0000144 played a carcinogenic role in PTC.

The ceRNA hypothesis suggests that circRNAs and mRNAs share miR response elements and competitively bind to these miRs [34]. Based on the hypothesis, we performed bioinformatic analysis and dual-luciferase reporter experiments to verify that circ_0000144 was a sponge of miR-1178-3p. Report of Wu et al. unmasked that miR-1178-3p targeted TLR4 to repress PTC cell malignancy [19, 20]. Our results exhibited lower levels of miR-1178-3p in PTC samples and cell lines, and miR-1178-3p inhibitor reversed circ_0000144 silencing-mediated impacts on PTC cell proliferation, apoptosis, migration, invasion, and PTC cell-induced angiogenesis, manifesting that circ_0000144 promoted malignant behaviors of PTC cells through sponging miR-1178-3p and repressing miR-1178-3p activity. Our results, in agreement with Wu et al. [19, 20], also suggested that miR-1178-3p had tumor-suppressive efficacy in PTC. However, miR-1178-3p promoted nasopharyngeal cancer [16] and pancreatic cancer [18] advancement by targeting STK4, p21, and CHIP, respectively, which might be related to the specificity of tumor tissue or cells.

Bioinformatic analysis and dual-luciferase reporter experiments further validated that YWHAH acted as a miR-1178-3p target. YWHAH is related to the pathogenesis of rheumatoid arthritis disease and schizophrenia [35, 36]. The upregulation of gremlin 1 played a role in human tumorigenesis through interaction with YWHAH [37]. Also, the combination of YWHAH knockdown and microtubule inhibitor was considered as a possible anti-cancer strategy [38]. In thyroid cancer, MAPKAPK5-AS1 facilitated cell malignancy by upregulating YWHAH through adsorbing miR-519e-5p, highlighting the oncogenic role of YWHAH [25]. In the current study, YWHAH was overexpressed in PTC samples and cell lines, and YWHAH elevation counteracted the inhibitory impact of miR-1178-3p mimic on PTC cell malignancy, indicating that YWHAH acted as an oncogene in PTC and miR-1178-3p mediated PTC progression by targeting YWHAH. Importantly, we observed that circ_0000144 elevated YWHAH expression through miR-1178-3p in PTC cells. Hence, we concluded that circ_0000144 functioned as a ceRNA and elevated YWHAH expression through sponging miR-1178-3p, thus promoting PTC progression.

## Conclusion

In sum, circ_0000144 played an oncogenic role in PTC. Mechanistically, circ_0000144 sponged miR-1178-3p to elevate YWHAH expression, resulting in facilitating PTC cell malignancy. The research provided evidence on circ_0000144 as a potential target for PTC treatment.

## Supplementary Information

**Additional file 1: Fig. S1**. **A** and **B** Relative expression of 3 miRs (miR-1178-3p, miR-485-3p, and miR-554) in PTC cells transfected with si-NC or si-circ_0000144. *P < 0.05 vs. si-NC. (C and D) Relative mRNA levels of 5 genes (DUSP4, YWHAH, IL17RD, PDPK1, and USP22) in PTC cells transfected with miR-NC or miR-1178-3p. *P < 0.05 vs. miR-NC.

## Data Availability

All data generated or analyzed during this study are included in this article.
