# Supplementary figures and images for: Circ_0000144 acts as a miR-1178-3p decoy to promote cell malignancy and angiogenesis by increasing YWHAH expression in papillary thyroid cancer
Source: J Otolaryngol Head Neck Surg. 2022 Jul 28;51:28. doi: 10.1186/s40463-022-00574-w (PMC9330660; doi:10.1186/s40463-022-00574-w)

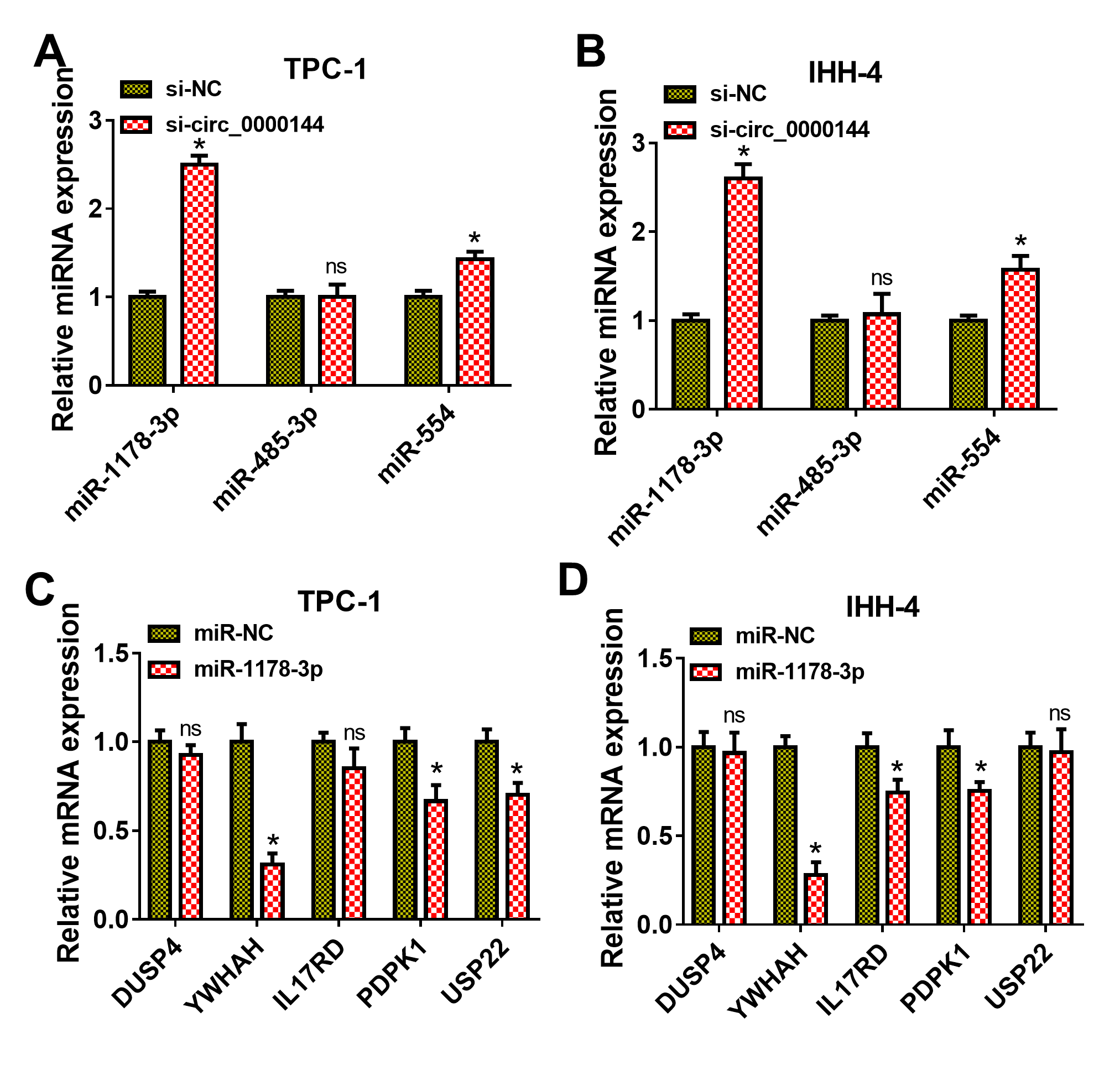

Supplement: Supplementary file 1 — Additional file 1: Fig. S1. A and B Relative expression of 3 miRs (miR-1178-3p, miR-485-3p, and miR-554) in PTC cells transfected with si-NC or si-circ_0000144. *P < 0.05 vs. si-NC. (C and D) Relative mRNA levels of 5 genes (DUSP4, YWHAH, IL17RD, PDPK1, and USP22) in PTC cells transfected with miR-NC or miR-1178-3p. *P < 0.05 vs. miR-NC. [file 40463_2022_574_MOESM1_ESM.tif]
